# When Clearing Out an Old House Advances Science: The Hump‐Shaped Diversity Distribution in Central European Grasslands Better Explained

**DOI:** 10.1111/gcb.70731

**Published:** 2026-02-02

**Authors:** Pascal Vittoz

**Affiliations:** ^1^ Institute of Earth Surface Dynamics, Faculty of Geosciences and Environment University of Lausanne Lausanne Switzerland

The proportion of natural grasslands in Central European landscapes prior to the Neolithic period, which were maintained through grazing by large herbivores or frequent disturbances, remains a subject of debate (Svenning 2002). However, most of the grasslands in the current landscape were created by deforestation following the Late Bronze Age (Hejcman et al. 2013). Centuries of stable management involving low‐intensity mowing or grazing without fertilisation have since created species‐rich ecosystems that are considered semi‐natural in Central Europe. Wilson et al. (2012) concluded that European temperate grasslands are the richest ecosystems in the world up to 50 m^2^, with a maximum of 43 vascular plant species on 0.1 m^2^ (in Romania) and 116 species on 25 m^2^ (in the Czech Republic).

As with many taxonomic groups, the species richness of vascular plants in Central European grasslands, when inventoried in plots, exhibits a hump‐shaped pattern in relation to elevation (e.g., Descombes et al. 2017). However, the reason for the lower species richness in lowland areas compared to mid‐elevations remains unclear. Until now, the hypothesis that grassland diversity decreased in low‐lying areas following agricultural intensification after the Second World War has largely been accepted (Descombes et al. 2017), despite a lack of available historical data to verify it. This intensification became possible following the production of mineral fertilisers after 1950, which allowed for a higher mowing frequency. Indeed, it is common to apply over 100 kg of nitrogen per hectare per year to meadows, with over three annual cuts (Zechmeister et al. 2003).

As Widmer et al. (2025) explained, there are numerous assessments of species richness evolution in temperate grasslands, with contrasting results. However, they are all based on quite recent baseline data, mostly from after 1970 that is, after the peak of agricultural intensification. Therefore, although a decrease in species richness at the plot level in these grasslands was strongly suspected, it has not yet been possible to quantify it.

In 2003, during a building renovation, some of the authors discovered boxes containing 580 exhaustive plant inventories of Swiss grasslands, recorded between 1884 and 1931 (Riedel et al. 2023). This impressive dataset was completely forgotten. The 0.3 × 0.3‐m plots were widely distributed across Switzerland, at elevations ranging from 212 to 2547 m.

Resurveying such small plots, with only rough location information (mostly the name of the village and the elevation), scattered in landscapes that have completely changed within a century, was challenging. The authors followed a convincing procedure to produce a comparable data set (Widmer et al. 2025): definition of a potential area based on available data and topographic maps; exclusion of areas outside grasslands; random selection of 3–5 plots in each potential area; resurvey of the selected new plots in the field. In total, the authors inventoried 1107 new plots in 277 potential areas, distributed between 322 and 2497 m.a.s.l. Although historical and resurveyed plots were not in exactly the same place, the mean Bray–Curtis dissimilarity between the resurveyed plots in a same potential area was on average 0.13 lower than that between historical and recent inventories. This shows that the plot location influences the species composition less than the time. The authors addressed the evolution of the α‐, β‐ and γ‐taxonomic diversity (species richness), the α‐ and β‐phylogenetic diversity, α‐ and β‐functional diversity (calculated from plant height, seed mass and specific leaf area), as well as the community‐weighted mean of functional traits and of ecological indicator values.

Plant ecologists familiar with the historical evolution of lowland ecosystems in relation to agriculture will not be surprised by the results. Nevertheless, they provide strong evidence of the losses suffered by Central European grasslands in the 20th century. On average, the resurveys contained 26% fewer species than the historical inventories, with the difference being particularly important in lowland areas (a 38% loss at around 500 m). β‐diversity decreased at all elevations, and γ‐diversity decreased by an average of 31%, approaching 50% around 1000 m. This species loss corresponded with a general decrease of the combined functional α‐diversity, as well as a 17% decrease in phylogenetic α‐diversity, with 17% fewer forbs, 42% fewer Cyperaceae and Juncaceae, but 47% more Poaceae (Figure 1). On average, the species present in the resurveys were more tolerant of disturbances and more competitive, but less tolerant of stress than those present in the historical surveys. The community‐weighted mean of ecological indicator values also suggested richer soils and higher tolerance of mowing. Almost all these differences decreased with elevation. Climate change induced an increase in the community‐weighted mean of the temperature ecological indicator value at all elevations, but the moisture levels remained unchanged.

**FIGURE 1:**
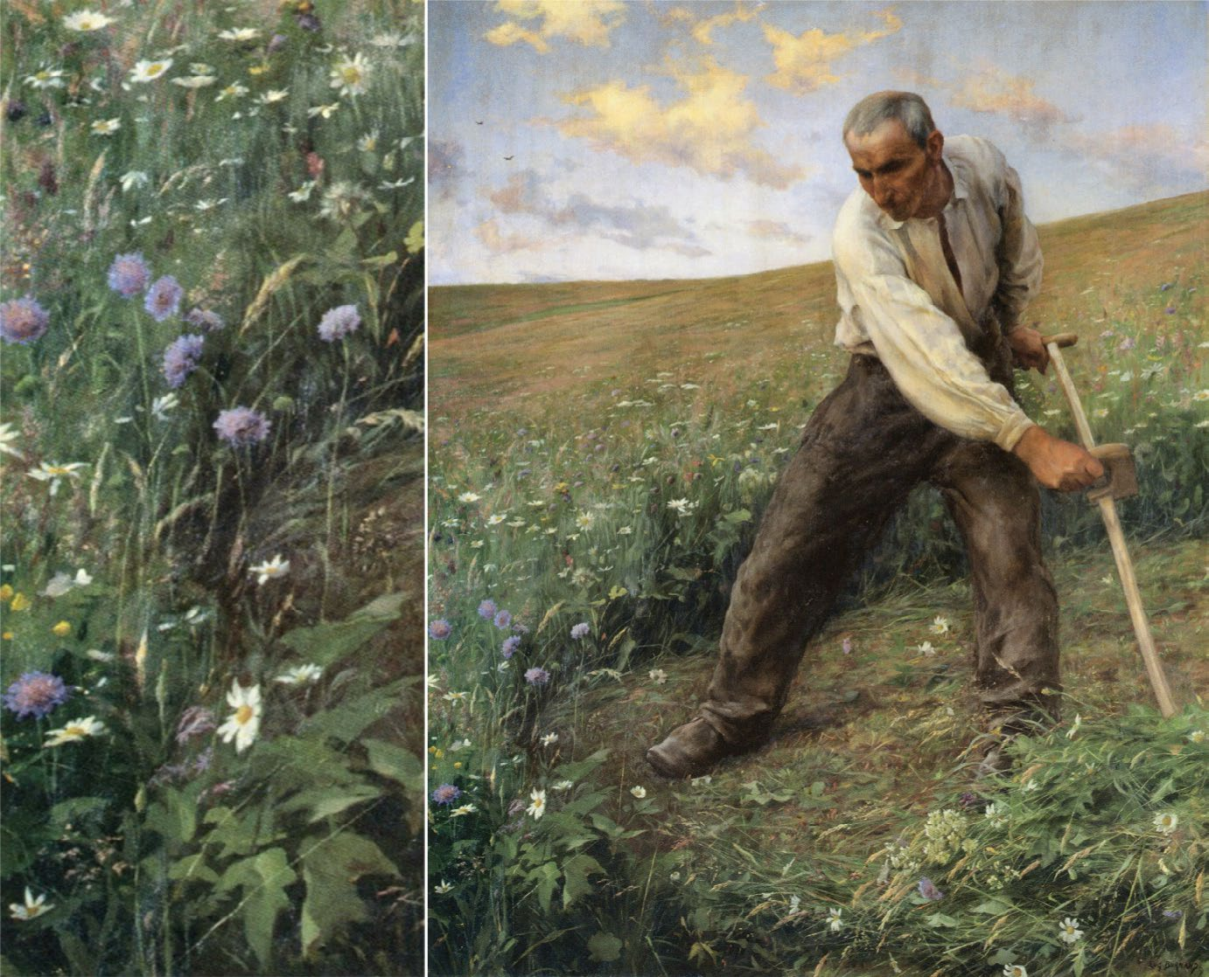
A species‐rich grassland in Western Switzerland in 1886. This painting by Eugène Burnand depicts the grass and forbs of a species‐rich grassland in great detail (see the enlarged bottom left corner of the painting). The high proportion of forbs, similar to those documented in historical records (Riedel et al. 2023; Widmer et al. 2025), is striking compared to lowland grasslands today.

Given the large set of analyses it contains and the long‐time frame, this study is probably one of the most comprehensive assessments of the evolution of diversity in European grasslands to be published to date. It provides a good understanding of the evolution of Swiss grasslands in the 20th century, which is likely to be similar to that in neighbouring countries. With these results, Widmer et al. (2025) quantified for the first time the substantial decline in species richness in grasslands following the intensification of agriculture, at both plot and landscape levels. Although the decrease in the lowlands was suspected, the extent of the impact at all elevations is surprising, especially the decrease in the proportion of forbs, which is independent of elevation. This suggests that mountain agriculture and subalpine summer pastures have not been completely preserved from the negative impacts of modernisation in agriculture. However, the greater reduction in species richness in the lowlands now clearly explains the hump‐shaped curve of species richness versus elevation in Central European grasslands: lowlands are poorer than the mid‐elevations because they have lost species. In an initial presentation of the historical dataset, Riedel et al. (2023) already demonstrated that plant species richness was not related to elevation. With 47 species in 0.09 m^2^, the richest plot was even richer than the published record in Romania (43 species in 0.1 m^2^; Wilson et al. 2012).

The consequences of intensification are not restricted to the diversity of vascular plants. The observed decline in forbs, which have largely been replaced by Poaceae, corresponds to a decrease in nectar production (see Figure 1), as well as a reduction in the diversity of forage for specialised insects. This is in addition to the direct destruction of invertebrates caused by mowing with modern machinery (Humbert et al. 2010). Both factors have an impact on the entire food web around grasslands. Furthermore, reduced phylogenetic diversity potentially weakens the general stability and resilience of grasslands.

Although agriculture was responsible for the losses, it cannot be blamed. Large parts of the European population were malnourished in the Second World War. Governments encouraged farmers to increase production using the newly and abundantly available fertilisers to improve countries' self‐sufficiency. In the following decades, the decrease in food costs, partly due to increased competition from imported agricultural products, put strong pressure on farmers to become highly efficient. Consequently, they either continued to fertilise grasslands intensively or they abandoned the difficult‐to‐manage ones (e.g., those in southern‐facing, steep areas) to forest recolonisation.

Species‐rich grasslands, on dry, oligotrophic slopes, are still existing in Central Europe. However, despite never being fertilised, they are poorer than they were one century ago (Riedel et al. 2023), and they have mostly been reduced to tiny, isolated patches surrounded by intensively exploited areas (1%–5% of their historical extent; Lachat et al. 2011; Loos et al. 2021). Hence, the small, remaining populations are threatened by low reproduction rates and inbreeding depression (Loos et al. 2021). Furthermore, the remaining species‐rich grasslands are under threat from nitrogen deposition, which in Central Europe mostly exceeds the critical load of 15 kg ha^−1^ year^−1^ (Roth et al. 2013). Therefore, it is crucial to monitor grasslands, particularly nutrient‐poor areas, to assess the long‐term effects of agricultural intensification on all trophic levels. The monitoring programme must be designed to distinguish between the respective contributions of inbreeding depression in small populations, nitrogen deposition, and climate change.

Supplementary projects would enhance our comprehension of the dynamics and consequences of these losses in grasslands. For example, Widmer et al. (2025) compared species richness in two time periods separated by over a century. Combining these results with those of similar shorter‐term studies should allow us to draw a precise curve showing how species have regressed over time. As in previous studies (e.g., Descombes et al. 2017), Widmer et al. (2025) calculated γ‐diversity as the total species richness of the plots in an elevational band. As the plots are small, this probably underestimates the real species richness in bands. Complementary data, such as historical flora or herbarium pieces, should be used to evaluate whether the actual γ‐diversity along the elevation gradient is similarly hump‐shaped, and to investigate how it evolved in the 20th century.

These historical datasets can be used to compile a list of the species that have declined the most. This enables conservation efforts to be focused on grasslands where these species are still abundant. However, what is certainly more important than anything else is that the remaining rich, nutrient‐poor grasslands must be reconnected. New oligotrophic grasslands must be (re)created quickly across the agricultural landscape, particularly in the lowlands where losses are highest, while high elevations must be preserved from intensification. Rebuilding an effective ecological infrastructure is essential, with large populations located close enough to each other to allow genetic exchange.

## Author Contributions

**Pascal Vittoz:** writing – original draft, writing – review and editing.

## Conflicts of Interest

The author declares no conflicts of interest.

## Linked Articles

This article is a Invited commentary on Widmer et al., https://doi.org/10.1111/gcb.70529.

## Data Availability

Data sharing not applicable to this article as no datasets were generated or analysed for the current article.
